# From Molecular Alterations to the Targeted Therapy: Treatment of Thalamic Glioma in Pediatric Patients

**DOI:** 10.3390/ijms27020695

**Published:** 2026-01-09

**Authors:** Yasin Yilmaz

**Affiliations:** Department of Pediatric Oncology, Istanbul Faculty of Medicine, Istanbul University, Istanbul 34390, Turkey; dryasinyilmaz@gmail.com

**Keywords:** thalamic glioma, low-grade glioma, high-grade glioma, molecular alterations, targeted therapy

## Abstract

Thalamic gliomas are among the most challenging pediatric brain tumors due to the delicate functions of the thalamus. Limited surgical intervention leads to the use of adjuvant therapies, including targeted therapy. Thalamic gliomas can be divided into two distinct groups: diffuse midline glioma (DMG) and low-grade glioma (LGG). The most common mutations that can be targeted for treatment are the KIAA1549-BRAF fusion; BRAF V600E mutation; EGFR, FGFR, PDGFR, NTRK, and CDK4/6 mutations; other MAP kinase pathway alterations; and PI3K/AKT/mTOR activation. The bithalamic high-grade glioma especially demonstrates EGFR mutations which makes it a distinct entity. Targeted therapy, including tyrosine kinas inhibitors has been shown to improve the overall survival compared to conventional therapy in certain situations. Demonstrating the mutation carried by the tumor is very critical in this regard. The purpose of this article is to focus on the treatment of thalamic glioma in pediatric patients in light of molecular information.

## 1. Introduction

Central nervous system (CNS) tumors constitute the most common group of solid tumors among childhood cancers. It is the second most frequent cancer type after leukemia. CNS tumors are the most common cause of cancer-related deaths in childhood [1].

The location and type of CNS tumors can vary depending on age. Supratentorial tumors are predominant in children under three years of age and over ten years of age, while infratentorial tumors are common in children between four and ten years of age [2].

CNS tumors are classified according to tumor classification guidelines established by the WHO, with the most recent classification made in 2021. According to this classification, molecular mutations have become important in diagnosis. Examples include diffuse midline glioma, H3K27-altered, and diffuse low-grade glioma, MAPK pathway-altered [3,4].

The incidence rate of primary CNS tumors in children was 5–7 per 100,000 and approximately 5000 children are diagnosed with CNS tumors annually in the United States [5].

Thalamic tumors account for approximately 1–5% of CNS tumors seen in childhood [6]. They may originate directly from the thalamus or from the point where it joins the cerebral peduncle (thalamopedicuncular) [7]. Due to its vital functions and proximity to critical tissues (hypothalamus, internal capsule, etc.), surgical intervention targeting the thalamus is often limited. However, advances in imaging techniques and intraoperative monitoring have enabled various surgical approaches, particularly for tumors without infiltrative characteristics [8,9,10]. As a result of studies on tumor genetics, molecular therapies are gaining importance in current treatment approaches.

**Figure 1:**
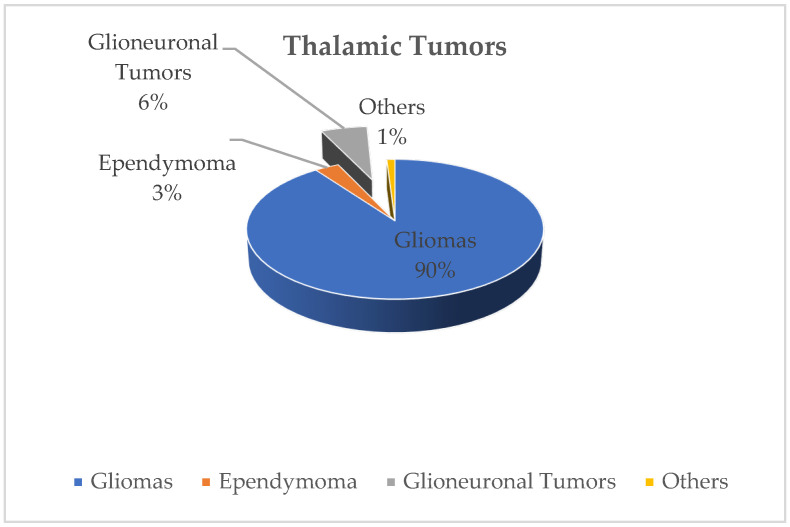
Distribution of thalamic tumors.

**Figure 2:**
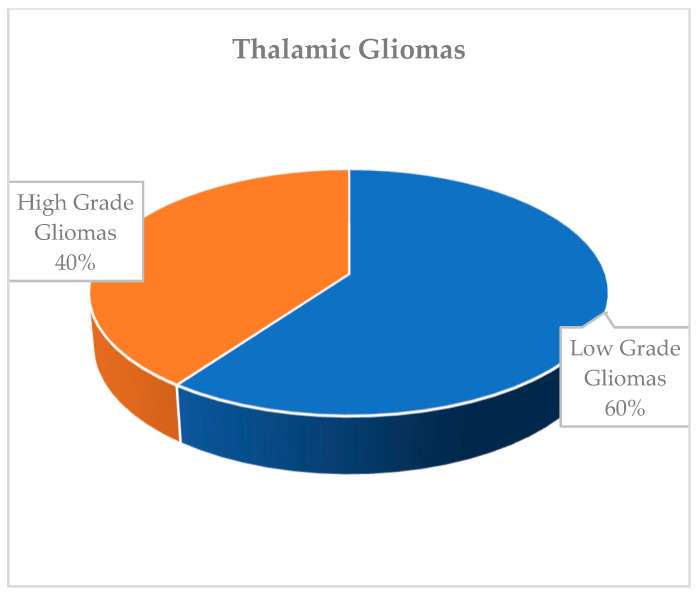
Distribution of thalamic gliomas.

Thalamic tumors are often unilateral (85%) and histopathologically consist of astrocytoma in 70–90% of cases [6]. Pilocytic astrocytoma and diffuse astrocytoma (grade II) constitute the majority of cases, making low-grade gliomas the most common thalamic tumors at a rate of 60%. High-grade thalamic gliomas (diffuse midline glioma) are seen at a rate of 40%. Ependymoma, glioneuronal tumors (ganglioglioma, neurocytoma, dysembryoplastic neuroepithelial tumor), and oligodendroglioma account for less than 10% (Figure 1 and Figure 2) [6].

Bithalamic tumors (15%) are rare and often exhibit diffuse histology. Due to factors such as surgical difficulty and the diffuse infiltrative histology of bithalamic tumors, the 10-year survival rate decreases to as low as 65%, while the survival rate for monothalamic tumors reaches up to 90% [11].

The imaging techniques can help in distinguishing the thalamic tumor as low- and high-grade. The high-grade gliomas exhibit larger volumes, greater heterogeneity in T2-weighted signal and enhancement, and lower minimum relative apparent diffusion coefficient (ADC) [12].

With the widespread use of NGS (Next Generation Sequencing) and WES (Whole Exome Sequencing) methods, genetic alterations are being detected at the tumor level. Targetable genetic alterations (mutations, amplifications, etc.) frequently reported for treatment purposes include BRAF, KIAA1549-BRAF fusion, FGFR, EGFR, NTRK, CDK4/6, and PDGFRA mutations. These genetic alterations, which can be seen in both low-grade and high-grade gliomas, may serve as alternatives or adjuncts to conventional chemotherapy and may have significant effects on survival [8,13].

The purpose of this article is to focus on the targeted therapy options in children with thalamic tumors. Since it has been previously shown that the use of tyrosine kinase inhibitor can improve overall survival, demonstrating a mutational map in tumors is crucial. This paper will summarize targeted treatment options along with tumor mutations.

## 2. The Neuroanatomy and Physiology of Thalamus

Thalamus is a major part of diencephalon that consists of mostly gray matter and a nuclear complex that consists of multiple nuclei. Its anatomical position is very critical because it is located between brainstem and cerebral cortex. It has many essential roles including relaying and integrating motor and sensory signals, regulation of consciousness, alertness, sleep, learning, and memory [14].

The right and left thalamic nuclei connect with each other via the interthalamic adhesion. It is in close proximity to the ventricles that form the upper and lateral walls of the third ventricle. It also has a close connection with part of the lateral ventricle floor and is very close to the basal ganglia [15].

The thalamus is essentially divided into three sections: the anterior, medial, and lateral thalamus, each of which contains approximately 30 different nuclei. The ventral anterior and ventral lateral nuclei are involved in motor cortex activities. The ventral posterior nucleus relay information to the primary somatosensory cortex. The medial segment of thalamus nuclei is responsible for integrating olfactory, somatic, and visceral afferent information. The anterior segment of the thalamus is especially integrated with limbic system and emotions [14,16].

## 3. Symptomatology in Thalamic Gliomas

Since the thalamus is a relay station and its anatomical position is of a great importance, symptoms related to invasion of the thalamus due to a tumor has a large spectrum. Two main mechanisms are playing a role in producing the symptoms in thalamic tumors. First, tumor-related local destruction of thalamic and adjacent tissue; secondly, elevation of intracranial pressure due to tumor mass [15]. These mechanisms can produce increased intracranial pressure (headache, dizziness, vomiting, and visual problems) and hydrocephalus, neuroendocrine dysfunction, seizure, motor and sensory deficits, gait disturbances, corticospinal signs, and extrapyramidal symptoms [2,17].

The rate of symptoms vary between studies and indicates hemiparesis (80%) and headache (50%) [18], hemiparesis (50%), symptoms of raised intracranial pressure (50%) and gait disturbance (15%) [8], motor deficit (70%) and altered sensorium (25%) [19], symptoms of raised intracranial pressure (65%) and limb weakness (45%) [20], symptoms of raised intracranial pressure (85%) and motor weakness, generally revealed by hemiparesis or gait disturbance (80%) [21], symptoms of increased intracranial pressure (55%), hemiparesis (20%), and tremor (10%) [17], and headache (30%) [22].

## 4. Thalamic Low-Grade Gliomas

Low-grade gliomas (LGG) are the most common (60%) type of thalamic glioma. They represent a heterogeneous group. Approximately half of LGGs are pilocytic astrocytomas (50%), while 25% have diffuse astrocytoma histopathology. More than half of monothalamic LGGs (55%) have pilocytic astrocytoma histopathology, while more than half of bithalamic LGGs (60%) exhibit diffuse astrocytoma histopathology [11].

The prognosis for thalamic LGGs is slightly lower compared to other localized LGGs in the central nervous system. The 10-year overall survival (OS) rate for all thalamic LGGs is 85–90%, the 10-year OS (overall survival) rate for monothalamic LGGs is 90–92%, while the OS rate for bithalamic LGGs is 65%. However, in terms of EFS (event free survival), the 10-year EFS rate for all thalamic LGGs is 29%, the 10-year EFS rate for monothalamic LGGs is 30%, while this rate drops to 15% for bithalamic LGGs [11]. These data indicate that the most important prognostic factor for thalamic LGGs is the location (monothalamic vs. bithalamic).

The EOR (extent of resection) rate in thalamic LGGs has been found to be associated with survival rate and EFS. While the EFS rate after total or subtotal resection is 50%, this rate drops to 20% after partial resection or biopsy. The 10-year OS rate for thalamic LGGs undergoing total resection is around 95%, while this rate drops to 85% for those undergoing partial resection or biopsy only. However, the gross total resection rate varies between 20 and 40% from center to center [8,11,18]. Partial resection can be performed in 20–30% of cases, while biopsy can be performed in 40–50% of patients, or no surgical procedure can be performed [11,19]. In over 80% of bithalamic LGGs, only a biopsy can be performed, and the partial resection rate remains very low.

Given such high survival rates, the low EFS rate indicates high recurrence and progression rates, suggesting that the remaining tissue after maximal safe surgery requires chemotherapy or radiotherapy. Thus, radiotherapy or chemotherapy is administered to patients with progressive symptoms or radiological progression.

**Figure 3:**
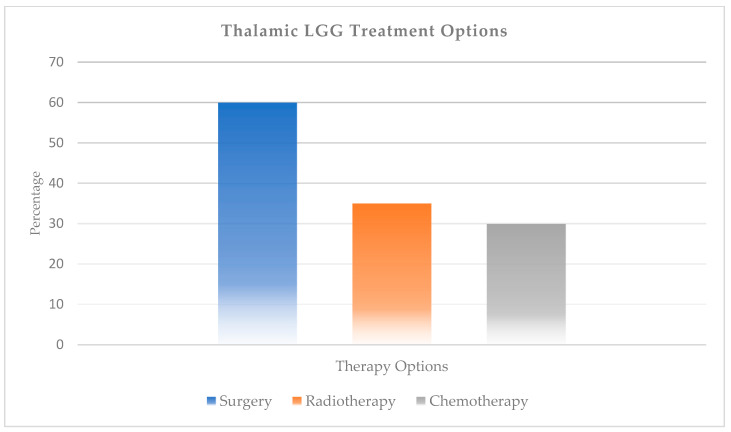
Therapy modalities for thalamic low-grade gliomas.

Approximately half of thalamic LGGs require non-surgical treatment (chemotherapy, radiotherapy). Although it varies from center to center, radiotherapy is used in 30–35% of patients, while chemotherapy is used in 25–30% of patients (Figure 3). The 10-year PFS (progression free survival) rate was 55% in patients who received radiotherapy as the first non-surgical treatment option, while it was 30–35% in patients who received conventional chemotherapy (containing vincristine and carboplatin). [11]. However, in long-term follow-up studies of patients who reached adulthood, OS rates were lower in patients who received radiotherapy compared to those who did not (77% vs. 97%) [23]. While less than 10% of bithalamic gliomas are amenable to surgical treatment, almost all are treated with radiotherapy and chemotherapy [24].

### Targeted Therapy for Thalamic LGG

**Figure 4:**
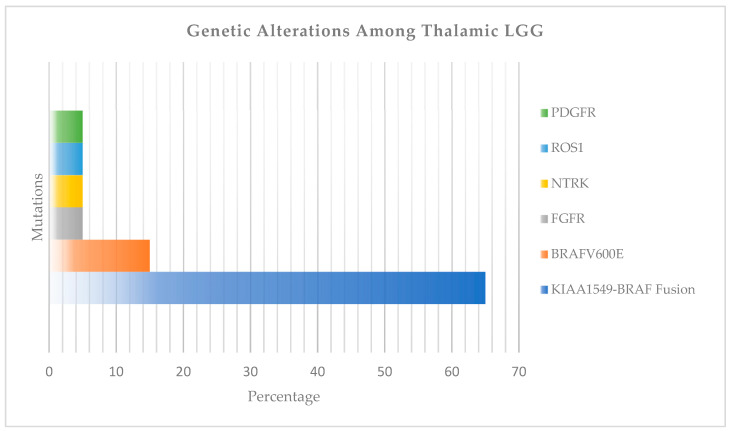
The most common genetic alterations among thalamic LGG.

Studies about molecular alterations in gliomas revealed that approximately 60–70% of thalamic LGGs carry the KIAA1549-BRAF fusion, and approximately 15% carry the BRAF V600E mutation [8,22]. Other receptor tyrosine kinase-related alterations (FGFR1, NTRK1, ROS1, PDGFR) account for nearly 15% (Figure 4) [8].

As in other CNS low-grade gliomas, BRAF alterations (mutation and fusion) are most commonly seen as molecular aberration in thalamic LGGs. BRAF mutation is clinically important since one of the main characteristics of gliomas harboring BRAF V600E mutations is poor response to the conventional chemotherapy and the risk of transforming to high-grade gliomas [25,26].

The first phase I/II study evaluating Trametinib with or without Dabrafenib in pediatric low-grade gliomas with BRAF V600 mutation, the PFS was reported higher (36.9 months) in the combination group compared to Trametinib monotherapy (16.4 months) [27]. In the above mentioned study, Trametinib monotherapy was less well-tolerated with 54% of patients discontinuing treatment for toxicity compared with 22% treated with combination (Trametinib plus Dabrafenib) therapy [27].

Dabrafenib in combination with Trametinib was approved by FDA for the treatment of LGG with BRAF V600E mutations in children [28]. Dabrafenib (C23H20F3N5O2S2) is a sulfonamide, a member of aminopyrimidine and a protein kinase inhibitor which selectively inhibits the B-RAF mutated protein. Trametinib (C26H23FIN5O4) is a pyridopyrimidine, a member of acetamides and a dual-kinase inhibitor which inhibits the mitogen-activated extracellular kinase 1 (MEK1) and MEK2. The overall response rate of combination therapy was strikingly high compared to conventional chemotherapy (47% vs. 11%). Moreover, the median PFS rate was longer in combination therapy than conventional therapy (20 months vs. 7.4 months). On the other hand, grade 3 and higher side effects were significantly lower in combination therapy than the conventional group (47% vs. 94%) [28].

Dabrafenib monotherapy is not recommended in LGG with BRAF fusion since there is a risk of causing paradoxical tumor growth. Tovorafenib (C17H12Cl2F3N7O2S) is a chloropyridine, a pan-RAF (A-Raf, B-Raf and C-Raf protein kinases) inhibitor, and a selective type II RAF kinase inhibitor which can be used as a monotherapy in both BRAF V600E mutations and BRAF fusions. A single-arm phase II clinical trial (FIREFLY-1) investigated the efficacy of Tovorafenib in children with relapsed or refractory LGG. The overall response rate was 51% and 42% of patients reported grade 3 or higher treatment-related adverse effect. However, decrease in growth velocity was reported (15%) in that study and, thus, patients should be carefully monitored during therapy in terms of height [29]. The another advantage of this drug might be availability of oral suspension form for younger children and lack of food effect. However, lack of standard arm (conventional therapy) is the main limitation of this trial and authors propose the phase III trial of Tovorafenib monotherapy versus conventional chemotherapy in the near future.

Selumetinib (C17H15BrClFN4O3) is a member of benzimidazoles and a strong inhibitor of mitogen-activated extracellular kinase pathway (MEK1 and 2). It was studied in children with recurrent/progressive LGG and a high rate of 2-year PFS was found (74%). Nearly 15% of children had LGG in thalamus and OS rate was 100% [30]. The study (PBTC-029B) was a prospective, multicenter phase 2 study, however sample size was relatively small and demonstrated significant results for non-NF1 patients.

Binimetinib (C17H15BrF2N4O3) is a member of benzimidazoles, another potent MEK inhibitor with a highly brain-penetrant character. It was studied in children with recurrent/progressive LGG with BRAF fusion. The overall response rate was found as 50% [31]. This was a phase II study and demonstrated significant results for LGG with and without BRAF fusion. Other medications (Ulixertinib, Plixorafenib) related to BRAF mutant gliomas were under investigation among pediatric populations [32,33]. One of the challenges encountered during the treatment of LGG with targeted therapy is tumor growth (rebound and/or regrowth) after cessation of therapy which might be because of the accumulation of an upstream activator [34]. One study interestingly reported a very high ratio (75%) of rapid progression after completion of MAPK inhibition which then gained good response after rechallenge [35]. Thus, terminology has been updated for rechallenge use of these targeted therapies. The rebound (rapid growth) is defined as >25% growth of an existing lesion within 3 months of cessation of systemic (MAPK inhibitor) therapy; recurrence regrowth is defined as >25% growth or a new lesion 6 months after stopping treatment as per RAPNO pLGG [36].

Beside BRAF pathway alterations (BRAF V600E mutation and BRAF fusion), receptor tyrosine kinases (RTKs)-related alterations have been identified in pediatric LGG, including FGFR (fibroblast growth factor receptor), NTRK (neurotrophic tropomyosin-related kinase), ROS (protein tyrosine kinase encoded by the ROS1 gene), ALK (anaplastic lymphoma kinase), and PDGFR (platelet-derived growth factor). The LGGs harboring these kinase fusions can respond to the targeted therapy in clinical conditions [37].

Debio1437 is an oral FGFR1-3 inhibitor that was used in recurrent/refractory pediatric LGG and HGG. A significant tumor reduction and partial response was obtained among five pediatric patients in a small single center study [38]. Erdafitinib (C25H30N6O2) is a potent FGFR1–4 tyrosine kinase inhibitor and was investigated its efficacy in pediatric low-grade glioma. The 6 month PFS was 45% and median duration of the stable disease was 6 months [39]. However, a recent study reported slipped capital femoral epiphyses in pediatric patients using FGFR inhibitors and thus the researcher emphasized that clinicians should closely monitor bone health during FGFR inhibitor usage [40]. This important side effect occurred both in the short term (5 months of usage) and long term (40 months of usage). Monitoring the short- and long-term side effects of tyrosine kinase inhibitors is very important and may sometimes lead to discontinuation of treatment.

Larotrectinib (C21H22F2N6O2) is a selective inhibitor of neurotrophin receptor kinase (NTRK) inhibitor approved by the FDA in 2018. A pediatric patient with recurrent thalamic low-grade glioma with NTRK2 fusion has been treated with Larotrectinib and near-complete resolution has been gained after 15-month use. No severe toxicity was observed and tolerated well in the pediatric patient [41]. Another report also demonstrated the stable disease without side effects in a child with LGG [42].

Everolimus (C53H83NO14) is a derivative of Rapamycin (sirolimus) and inhibits the activation of the mammalian target of Rapamycin (mTOR), which can be used for low- and high-grade gliomas. The phosphatidylinositol 3-kinase (PI3K), AKT, and mammalian target of Rapamycin (mTOR) activation can be seen in up to 50% of pediatric LGGs [43]. The PNOC001 phase II single-arm trial investigated the efficacy of Everolimus for progressive/recurrent pLGGs. A proportion of 60% of patients had PI3K/AKT/mTOR activation. The 6-month PFS was 67.4% and median PFS was 11.1 months. Researchers, importantly, reported that novel KIAA1549::BRAF fusion breakpoints were most common in supratentorial midline pilocytic astrocytoma and correlated with poor clinical outcomes. However, they emphasized that PI3K/AKT/mTOR pathway activation was not found to be related to OS and median PFS. Thus, authors suggested that PI3K/AKT/mTOR activation might not predict Everolimus response in recurrent LGGs [44].

## 5. Thalamic High-Grade Gliomas

**Figure 5:**
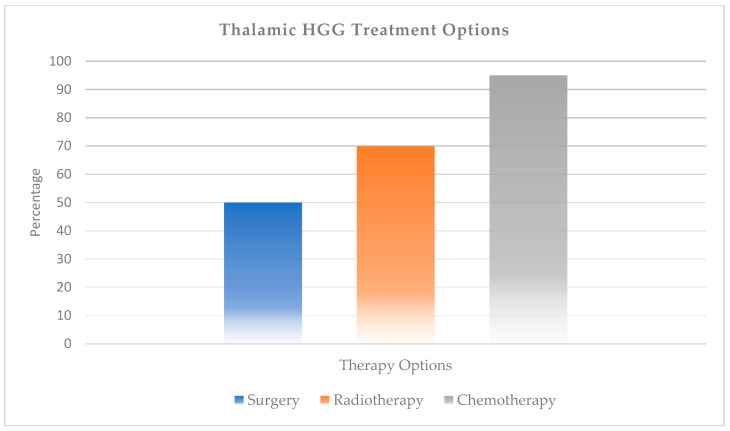
Therapy modalities for thalamic high-grade gliomas.

Thalamic high-grade gliomas (diffuse midline glioma originating from the thalamus) account for 15% of pediatric HGGs and 40% of thalamic gliomas [45]. Gliomas located in the thalamopulvinar region have been reported to be associated with high-grade, H3K27 mutation, and poor outcome [46]. In thalamic HGG, as in LGG, EOR (extent of resection) has been found to be associated with survival [47]. Maximal safe surgical resection followed by focal radiotherapy and chemotherapy is the current standard therapy for pHGG. However, the subtotal/partial resection rate in thalamic HGGs does not exceed 50% (Figure 5) [20,45]. Factors affecting prognosis in thalamic gliomas were found to be maximal safe surgery (any resection > 50%) and histopathology (LGG vs. HGG) [48]. The ORR (<20%) and 2-year survival rate (<35%) is significantly low in pediatric HGG compared to LGG. Thus, chemotherapy is frequently administered to almost all thalamic HGGs [19].

H3K27 mutations were detected in 70% of monothalamic high-grade gliomas, whereas this mutation was rarely detected in bithalamic gliomas [24,47]. This feature also demonstrates that bithalamic gliomas possess unique characteristics. It has been reported that more than 90% of bithalamic gliomas show high-grade glioma histopathology.

PDGFRA amplification was detected in less than 20% of bithalamic HGGs, EGFR mutation in 10%, and CDK4/6 amplification in 10% in bithalamic HGGs [24]. Since the number of patients with bithalamic HGGs in the studies is very small, the rate of molecular mutations varies in a large range. Thus, another publication reported an EGFR mutation rate of 85% in bithalamic gliomas [49]. In the first study, only one patient (10%) had EGFR mutation (EGFR G598V) among ten patients, and this mutation is a classically detected mutation that occurs in the extracellular domain of the EGFR protein. However, the latter study demonstrated EGFR mutations in eleven patients (85%) out of thirteen patients, and nine of these EGFR mutations (80%) were small in-frame insertions within exon 20 which is part of the intracellular domain of the EGFR protein. And the author suggested that these insertions were not reported before in pediatric high-grade gliomas.

The absence of H3K27 mutations and the presence of EGFR mutations in bithalamic high-grade gliomas suggest that they may be a distinct entity.

The presence of EGFR mutations is particularly noteworthy in thalamic HGG, especially in bithalamic HGG [47,49,50,51]. The EGFR alteration rate (mutation, amplification) among all thalamic HGG (monothalamic plus bithalamic) is around 30–40% [52]. The use of tyrosine kinase inhibitors such as Osimertinib, Afatinib, and Trametinib, which target this mutation, facilitates treatment [49,53]. In bithalamic gliomas with EGFR mutation, patients treated with TKIs (tyrosine kinase inhibitors) had a higher overall survival (OS) rate compared to those receiving conventional chemotherapy [50].

The TP53 mutation is observed in 60% of thalamic HGGs and is associated with poor prognosis [20]. The CDK4/6 mutation can be observed in 15% of bithalamic HGGs [49].

### Targeted Therapy for Thalamic HGG

ONC201 is a first-in-class small molecule that antagonizes the G protein-coupled receptor (GPCR) and dopamine receptors D2 (DRD2) that causes p53-independent apoptosis in tumor cells via AKT/ERK inactivation [54]. The efficacy of the ONC201 drug (Dordaviprone) in patients with H3K37 mutations among thalamic HGGs has been investigated and shown to provide better OS compared to brainstem-origin HGGs. Furthermore, it has been shown that reirradiation after initiation of ONC201 treatment improves survival [54]. This study was a result of a multicenter (across 14 European countries) developed study. The median OS since diagnosis was nearly 15 months which is comparable for DMGs. Thalamic HGG was found to be a favorable prognostic factor among other DMGs. With its easy use once a week, ONC201 appears to be an important agent in the near future. Long-term survival has also been demonstrated in thalamic HGG patients using ONC201 [55].

Among DMG (diffuse midline glioma) patients with the H3K27 mutation, a 20% rate of MAPK pathway alterations has been reported, and 60% of these alterations were observed in thalamic-origin HGGs. The BRAFV600E mutation was detected in 10% of thalamic-origin HGGs.

The combination of Dabrafenib and Trametinib has been investigated among pediatric HGG patients harboring BRAF V600E mutation. The overall response rate (ORR) was found as 56%, median duration of response (DOR) was found as 22.2 months, and median OS was found as 32.8 months [56]. This was a phase II trial and included a relatively small sample size. Almost all patients received radiotherapy and miscellaneous chemotherapy before using Dabrafenib and Trametinib. Because of the lack of a randomized comparison, results were compared to historical cohorts treated with traditional chemotherapy. And unfortunately, half of the patients discontinued treatment due to progressive disease. Nevertheless, results were promising for HGGs with BRAF V600E mutations which are refractory to conventional treatments.

A retrospective analysis in pediatric patients with newly diagnosed nineteen BRAF V600–mutant HGG who were treated with BRAF inhibitors with (60%) or without (40%) MEK inhibitors suggested improved outcomes compared to those who were treated with conventional chemotherapy [57]. In that analysis, 3-year PFS was found as 65% and OS was 82%. Note that three-year OS was around 40% in patients treated with conventional therapy. There were no significant differences found between monotherapy and combination therapy in terms of survival outcomes. Note that although all of the patients in that cohort had BRAF V600E mutation, only four of them had the H3K27 mutation.

**Figure 6:**
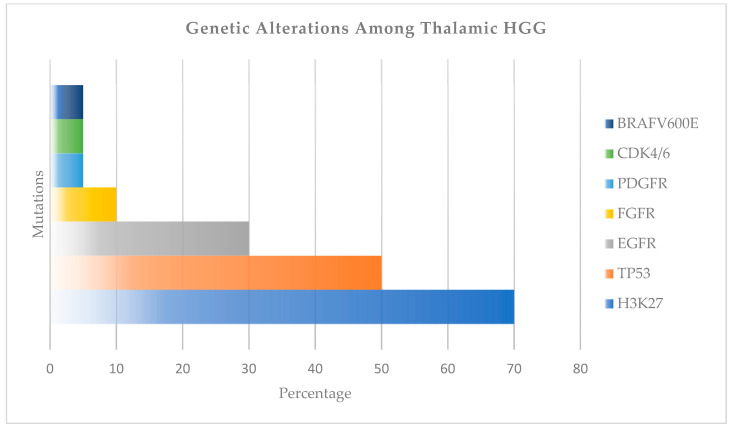
The most common genetic alterations among thalamic HGG.

Other mutations include PDGFRA, NRAS, and FGFR mutations, which were detected at a frequency of 10% (Figure 6) [58,59]. The use of Erdafitinib for FGFR mutation is reported in the literature. Imatinib (C29H31N7O), a specific tyrosine kinase receptor inhibitor, Dasatinib (C22H26ClN7O2S), a multi-kinase inhibitor for PDGFR inhibition, Vandetanib (C22H24BrFN4O2), a multi-kinase inhibitor for VEGFR2 inhibition, Gefitinib (C22H24ClFN4O3), a selective tyrosine kinase receptor inhibitor, and Erlotinib (C22H23N3O4), a tyrosine kinase receptor inhibitor for EGFR inhibition, were trialed previously and reported in the literature [60].

Nimotuzumab is an Anti-EGFR antibody and has been especially investigated in DIPG [61]. The similar mechanism can suggest its usage in thalamic high-grade glioma with no major adverse effect. This monoclonal antibody is commonly used in combined regimens in CNS high-grade gliomas, even concomitant to radiotherapy [62]. A recent study investigated the efficacy of radiotherapy for concomitant and adjuvant Nimotuzumab/Vinorelbine combination in patients with thalamic high-grade gliomas. The PFS rate was 33.3% at one year and the OS was 88.9%, 33.3%, and 22.2% at 1, 2, and 3 years [63]. Although this study used a very small sample size (five thalamic HGG and four other non-DIPG DMGs), the study was designed very homogenously as all patients were treated with radiotherapy and concomitant/adjuvant Nimotuzumab/Vinorelbine. The results were comparable with those which patients with DIPG already had. Studies in which patients are treated homogeneously, such as this study, will yield more meaningful results in terms of OS and PFS.

PDGFRA alterations (mutation and amplifications) can be seen in pediatric thalamic high-grade gliomas. The tyrosine kinase inhibitors (TKI) that can target PDGFR signaling are Imatinib and Dasatinib. Since the pediatric high-grade gliomas carrying PDGFRA alteration are associated with worse outcome, TKI can be used among these patients to prolong the survival [64]. Dasatinib is an oral tyrosine kinase inhibitor with a stronger inhibition of PDGFR signaling than earlier generation TKIs.

ACVR mutations can be seen in pediatric high-grade gliomas. Although there are no ACVR1 inhibitors clinically in use, Vandetanib, an inhibitor of VEGFR/RET/EGFR, was found to target ACVR1. Although the patients treated with Vandetanib plus Everolimus were not thalamic DMG (they were DIPG), the clinical use of this drug might be promising for future clinical trials [65].

The PI3K/AKT/mTOR pathway regulates the cell cycle and is commonly dysregulated in diffuse midline gliomas. Everolimus, a Rapamycin analog, has been investigated in DMG in combination with Vandetanib and PDGFR inhibitors [60]. Perifosine (C25H52NO4P) is a third generation oral alkylphospholipid targeting Akt’s pleckstrin domain, causing inhibition of Akt phosphorylation [66]. Perifosine was used in pediatric patients with high-grade gliomas; although well-tolerated, it unfortunately did not show any improvement in OS or PFS [66].

**Figure 7:**
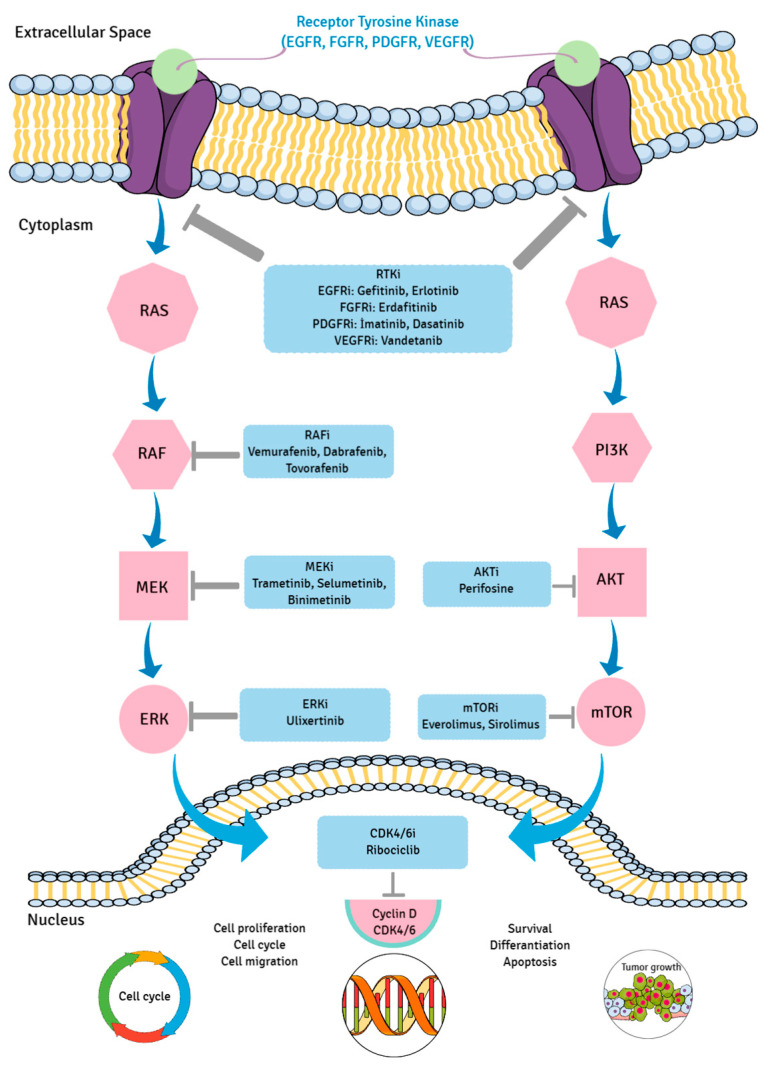
The targeted pathway for mitogen-activated protein kinase (MAPK) cascades and inhibitors is illustrated in this figure. RTKi: receptor tyrosine kinase inhibitor; EGFRi: epidermal growth factor receptor inhibitor; FGFRi: fibroblast growth factor receptor inhibitor; PDGFRi: Platelet-derived growth factor receptor inhibitor; VEGFRi: vascular endothelial growth factor receptor inhibitor; RAFi: RAF inhibitor; MEKi: MEK inhibitor; ERKi: ERK inhibitor; AKTi: AKT inhibitor; mTORi: mTOR inhibitor; CDK4/6i: CDK4/6 inhibitor.

Ribociclib (C23H30N8O) is a selective cyclin-dependent kinase inhibitor which specifically inhibits CDK4 and CDK6 and was investigated in DMG. Although the improvement was not demonstrated in the phase 1 trials [67], the combination therapy of Ribociclib and Everolimus is promising for future studies in pediatric patients with DMGs. Cases of thalamic HGG without detected mutations treated with Temozolomide have also been reported in the literature [19]. The available targeted therapy drugs for thalamic gliomas are listed in Table 1 and MAP kinase pathway was illustrated in Figure 7.

The new methods are increasing to treat the pediatric high-grade gliomas, especially for diffuse midline gliomas. Since the cell surface of diffuse midline gliomas highly express disialoganglioside GD2, immunotherapy targeting GD2 has been began use among patients with DMG. The chimeric antigen receptor (CAR) T cell studies have shown efficacy against DMG targeting GD2. However, neurotoxicity was a significant side effect [68]. The most recent technique, transient CAR T cells targeting GD2 created with mRNA, was successfully utilized in thalamic DMG xenograft model without causing neurologic toxicity [69]. Results are promising for thalamic HGGs in the near future. However, concerns about neurotoxicity raise ethical and safety issues in the pediatric population; therefore, more studies in vitro and in adults are needed before this type of treatment can be used in children.

## 6. Conclusions

In the treatment of thalamic gliomas, due to the inability to perform surgery at the desired level because of the anatomical proximity to vital tissues, the high rates of recurrence and progression, even in cases where surgery is possible, and the low EFS, the addition of molecular investigations to standard follow-up has become the current follow-up approach.

In high-grade gliomas with a defined mutation (e.g., BRAF mutation, EGFR mutation), patients receiving targeted therapy (tyrosine kinase inhibitor) had a higher overall survival (OS) rate compared to those receiving conventional chemotherapy [50]. Targeted therapies are becoming more widespread due to reasons such as increased survival rates, lower toxicity profiles, and ease of use. Among targeted therapies, B-RAF inhibitor and MEK inhibitors are the most commonly used. The use of tyrosine kinase inhibitors (TKI) has been shown to increase the survival rate and overall response rate compared to conventional chemotherapy in pediatric high- and low-grade gliomas [70]. However, the mechanism of TKI resistance, treatment duration, and long-term side effects remain issues that need to be clarified. In addition to growth velocity, all parameters related to growth and development should be monitored during targeted therapy, as unexpected side effects may occur in the long term.

As studies aimed at detecting molecular changes increase, opportunities for targeted therapies will arise, and we believe these opportunities will contribute to both patient survival rates and increased EFS rates. Prospective and multi-institutional studies involving long-term observations are strongly needed in order to make definitive and consistent conclusions.

## Figures and Tables

**Table 1:** Available targeted therapy drugs for thalamic gliomas.

Name of Drug	Category
Perifosine	AKT Inhibitor
Dabrafenib	B-RAF Inhibitor
Ribociclib	CDK4/6 Inhibitor
Dordaviprone	DRD2 Inhibitor
Gefitinib	EGFR Inhibitor
Erlotinib	EGFR Inhibitor
Nimotuzumab	EGFR Inhibitor
Erdafitinib	FGFR1-4 Inhibitor
Trametinib	MEK1/2 Inhibitor
Selumetinib	MEK1/2 Inhibitor
Binimetinib	MEK1/2 Inhibitor
Everolimus	mTOR Inhibitor
Larotrectinib	NTRK Inhibitor
Tovorafenib	Pan-RAF Inhibitor
Imatinib	PDGFR Inhibitor
Dasatinib	PDGFR Inhibitor
Vandetanib	VEGFR Inhibitor

## Data Availability

No new data were created or analyzed in this study. Data sharing is not applicable to this article.

## Abbreviations

ACVR	Activin A receptor
ADC	Apparent diffusion coefficient
AKT	Protein kinase B
ALK	Anaplastic lymphoma kinase
BRAF	B-rapidly accelerated fibrosarcoma
CAR	Chimeric antigen receptor
CDK	Cyclin-dependent kinase
CNS	Central nervous system
DIPG	Diffuse intrinsic pontine glioma
DMG	Diffuse midline glioma
DOR	Duration of response
DRD2	Dopamine receptors D2
EFS	Event free survival
EGFR	Epidermal growth factor receptor
EOR	Extent of resection
FDA	Food and Drug Administration
FGFR	Fibroblast growth factor receptor
GD2	Disialoganglioside
LGG	Low-grade glioma
MAPK	Mitogen-activated protein kinase
MEK	Mitogen-activated extracellular kinase
mTOR	Mammalian target of Rapamycin
NGS	Next Generation Sequencing
NRAS	Neuroblastoma RAS viral oncogene homolog
NTRK	Neurotrophic tropomyosin-related kinase
OS	Overall survival
PA	Pilocytic astrocytoma
PDGFR	Platelet-derived growth factor receptor
PI3K	Phosphatidylinositol 3-kinase
RAPNO	Response Assessment in Pediatric Neuro-Oncology
RET	Rearranged during transfection
RTK	Receptor tyrosine kinase
WES	Whole Exome Sequencing
